# A Retrospective Analysis of Thrombosis and COVID-19 Mortality in Rural Midwestern Population

**DOI:** 10.7759/cureus.74320

**Published:** 2024-11-23

**Authors:** John Dunton, Kaitlin Bierman-Macke, Taylor Little, Nicholas Zuk, Nova Beyersdorfer, Scott Goade, Kerry Johnson, Greg Stahl, Robert D Arnce

**Affiliations:** 1 Emergency Medicine, Kansas City University, Kansas City, USA; 2 Primary Care Medicine, Kansas City University, Joplin, USA; 3 Clinical Pharmacy, Freeman Health System, Joplin, USA; 4 College of Medicine, Kansas City University, Joplin, USA; 5 Mathematics, Missouri Southern State University, Joplin, USA; 6 Statistics, Freeman Health System, Joplin, USA; 7 College of Osteopathic Medicine, Kansas City University of Medicine and Biosciences, Joplin, USA

**Keywords:** corona virus disease 2019 (covid-19), covid-19-associated coagulopathy, missouri, rural hospital, thrombosis, thrombosis and covid-19 mortality in missouri

## Abstract

Background

COVID-19 disease has caused a major global impact on health and mortality. This infection may predispose patients to thrombotic disease, caused by excessive inflammation, endothelial dysfunction, platelet activation, and stasis. In this study, we compared mortality rates in patients admitted to the hospital with the diagnosis of COVID-19, who also had the additional diagnosis of thrombosis with those who did not have thrombosis as an additional diagnosis.

Methods

This retrospective observational study compared mortality rates in patients admitted to the hospital with the diagnosis of COVID-19, with and without thrombosis, as well as those patients admitted to the hospital with the diagnosis of thrombosis who did not have COVID-19. The diagnoses were verified using International Classification of Diseases Tenth Revision (ICD-10) codes, a standard among electronic medical records (EMR). The data were taken from the EMR at Freeman Health System in Joplin and Neosho, Missouri, from April 2020 to December 2021. This patient population is representative of not only Southwest Missouri but also the four-state area, including Oklahoma, Arkansas, and Kansas. The ICD-10 codes were used to separate the patient population into three main groups as follows: patients diagnosed with COVID-19 without thrombosis, patients diagnosed with thrombosis without COVID-19, and patients diagnosed with both COVID-19 and thrombosis. These three categories were then subdivided by age and biological sex. Sample proportions were completed using Wald's method, and the two-sample proportion summary hypothesis test with confidence intervals was used for the proportion difference.

Results

A total of 3,094 patients were included in the study population. Excluded from the study were patients who were previously admitted to a hospital for COVID-19 and duplicate admissions. The mortality rate was highest (0.4714) in patients concurrently diagnosed with COVID-19 and thrombosis (Population 1 {P1}), followed by patients diagnosed with COVID-19 without thrombosis (Population 2 {P2}, 0.1187). Patients diagnosed with thrombosis without COVID-19 (Population 3 {P3}, 0.1216) had the lowest mortality. Two sample proportion hypothesis tests determined confidence intervals (CI) for mortality risk comparing P3 to P1 (95% CI: 0.2888-0.4108, p<0.0001) and P2 to P1 (95% CI: 0.2919-0.4135, p<0.0001).

Discussion

In this rural, Midwestern population, patients admitted to the hospital with the diagnosis of COVID-19 and thrombosis had significantly increased mortality rates compared to patients admitted with the diagnosis of COVID-19 or thrombosis alone.

Conclusion

The data from this study indicated that individuals diagnosed with both COVID-19 and thrombosis had a higher likelihood of mortality compared to those diagnosed with COVID-19 without thrombosis and those diagnosed with thrombosis without COVID-19. This information could assist physicians in determining treatment plans for patients diagnosed with COVID-19 and a secondary complication of thrombosis.

## Introduction

The Centers for Disease Control and Prevention (CDC) defines COVID-19 as a respiratory disease caused by the SARS-CoV-2 virus, a coronavirus, which was discovered in 2019 [1]. SARS-CoV-2 is an enveloped positive-sense single-stranded RNA (+ssRNA) virus spread through human interaction and respiratory material (e.g., cough or sneeze) via infected individuals [2]. Since the World Health Organization (WHO) declared the COVID-19 outbreak a pandemic on March 11, 2020, there have been 6.9 million deaths to date [3,4]. The symptoms and severity of COVID-19 infection are widely varied. It is estimated that 33% of infected people never develop symptoms, while others suffer severe morbidity and mortality [5]. The case-fatality rate for COVID-19 differs by age. In the United States, patients aged five to 17 years had 0.3 deaths per 1,000 cases. In comparison, 304.9 deaths per 1,000 cases occurred among patients aged 85 years or older. Among patients hospitalized in intensive care, the case fatality rate is 40% [5]. Several complications secondary to COVID-19 infections include respiratory failure, cardiac and cardiovascular complications, neurologic complications, inflammatory complications, secondary infections, and our main topic of discussion, thromboembolic complications [6].

COVID-19 can predispose individuals to venous or arterial thrombosis due to the presence of increased endothelial damage, inflammatory response, hypoxia, hyper-viscosity, and immobilization [7,8]. Approximately 20-50% of hospitalized COVID-19 patients exhibit hematologic changes in coagulation studies, which are initially characterized more by thrombotic events than by hemorrhagic complications. Notably, up to 71.4% of COVID-19-related deaths occur in patients with disseminated intravascular coagulation (DIC) [7]. Patients with COVID-19 can present with coagulation abnormalities that mimic other systemic coagulopathies associated with severe infections, these include DIC or thrombotic microangiopathy. Prior studies have shown that coagulopathy in patients with COVID-19 increases the risk of death [9]. This study aimed to improve our understanding of COVID-19 and secondary COVID-19-related thrombosis by assessing the mortality rate of hospitalized patients with COVID-19 and thrombosis.

This article was previously presented as a live presentation at the Kansas City University Research Symposium on April 5, 2023.

## Materials and methods

This retrospective clinical study was performed on data collected at Freeman Health System (FHS) located in Southwest Missouri. FHS is one of the predominant hospital systems in Southwest Missouri, also serving parts of Kansas, Arkansas, and Oklahoma. The data were extracted from the electronic medical records (EMR) of patients admitted to the hospital from April 1, 2020, to December 31, 2021. All participants were aged 18 years or older. Since this is a retrospective observational study, individual informed consent was not required.

Hospitalized patients were then stratified based on the presence or absence of COVID-19 infection and/or thrombosis as a diagnosis in their EMR. To be included in the study, the patient had to be diagnosed with thrombosis, COVID-19, or COVID-19 with thrombosis. The specific ICD-10 codes used can be seen in Table 1. The ICD-10 codes were chosen to give the largest and most specific patient population base for this study.

**Table 1:** COVID-19 and thrombosis diagnostic codes. ICD-10: International Classification of Diseases Tenth Revision

ICD-10 code	Diagnosis
U071	COVID-19
D473	Essential (hemorrhagic) thrombocythemia
D6859	Other primary thrombophilia
D6869	Other thrombophilia
D693	Immune thrombocytopenic purpura
D6942	Congenital and hereditary thrombocytopenia purpura
D6949	Other primary thrombocytopenia
D6959	Other secondary thrombocytopenia
D696	Thrombocytopenia, unspecified
D7582	Heparin-induced thrombocytopenia (HIT)
I513	Intracardiac thrombosis, not elsewhere classified
I63311	Cerebral infarction due to thrombosis of right middle cerebral artery
I63312	Cerebral infarction due to thrombosis of left middle cerebral artery
I742	Embolism and thrombosis of arteries of the upper extremities
I743	Embolism and thrombosis of arteries of the lower extremities
I745	Embolism and thrombosis of iliac artery
I81	Portal vein thrombosis
I82401	Acute embolism and thrombosis of unspecified deep veins of right lower extremity
I82402	Acute embolism and thrombosis of unspecified deep veins of left lower extremity
I82403	Acute embolism and thrombosis of unspecified deep veins of lower extremity, bilateral
I82411	Acute embolism and thrombosis of right femoral vein
I82412	Acute embolism and thrombosis of left femoral vein
I82431	Acute embolism and thrombosis of right popliteal vein
I82432	Acute embolism and thrombosis of left popliteal vein
I82441	Acute embolism and thrombosis of right tibial vein
I82442	Acute embolism and thrombosis of left tibial vein
I82451	Acute embolism and thrombosis of right peroneal vein
I82452	Acute embolism and thrombosis of left peroneal vein
I82462	Acute embolism and thrombosis of left calf muscular vein
I824Z3	Acute embolism and thrombosis of unspecified deep veins of distal lower extremity, bilateral
I82512	Chronic embolism and thrombosis of left femoral vein
I825Y3	Chronic embolism and thrombosis of unspecified deep veins of proximal lower extremity, bilateral
I82611	Acute embolism and thrombosis of superficial veins of right upper extremity
I82612	Acute embolism and thrombosis of superficial veins of left upper extremity
I82613	Acute embolism and thrombosis of superficial veins of upper extremity, bilateral
I82619	Acute embolism and thrombosis of superficial veins of unspecified upper extremity
I82621	Acute embolism and thrombosis of deep veins of right upper extremity
I82622	Acute embolism and thrombosis of deep veins of left upper extremity
I82623	Acute embolism and thrombosis of deep veins of upper extremity, bilateral
I82812	Embolism and thrombosis of superficial veins of left lower extremity
I82A11	Acute embolism and thrombosis of right axillary vein
I82A12	Acute embolism and thrombosis of left axillary vein
I82B11	Acute embolism and thrombosis of right subclavian vein
I82C11	Acute embolism and thrombosis of right internal jugular vein
T82867A	Thrombosis due to cardiac prosthetic devices, implants and grafts, initial encounter
T82868A	Thrombosis due to vascular prosthetic devices, implants and grafts, initial encounter

**Figure 1:**
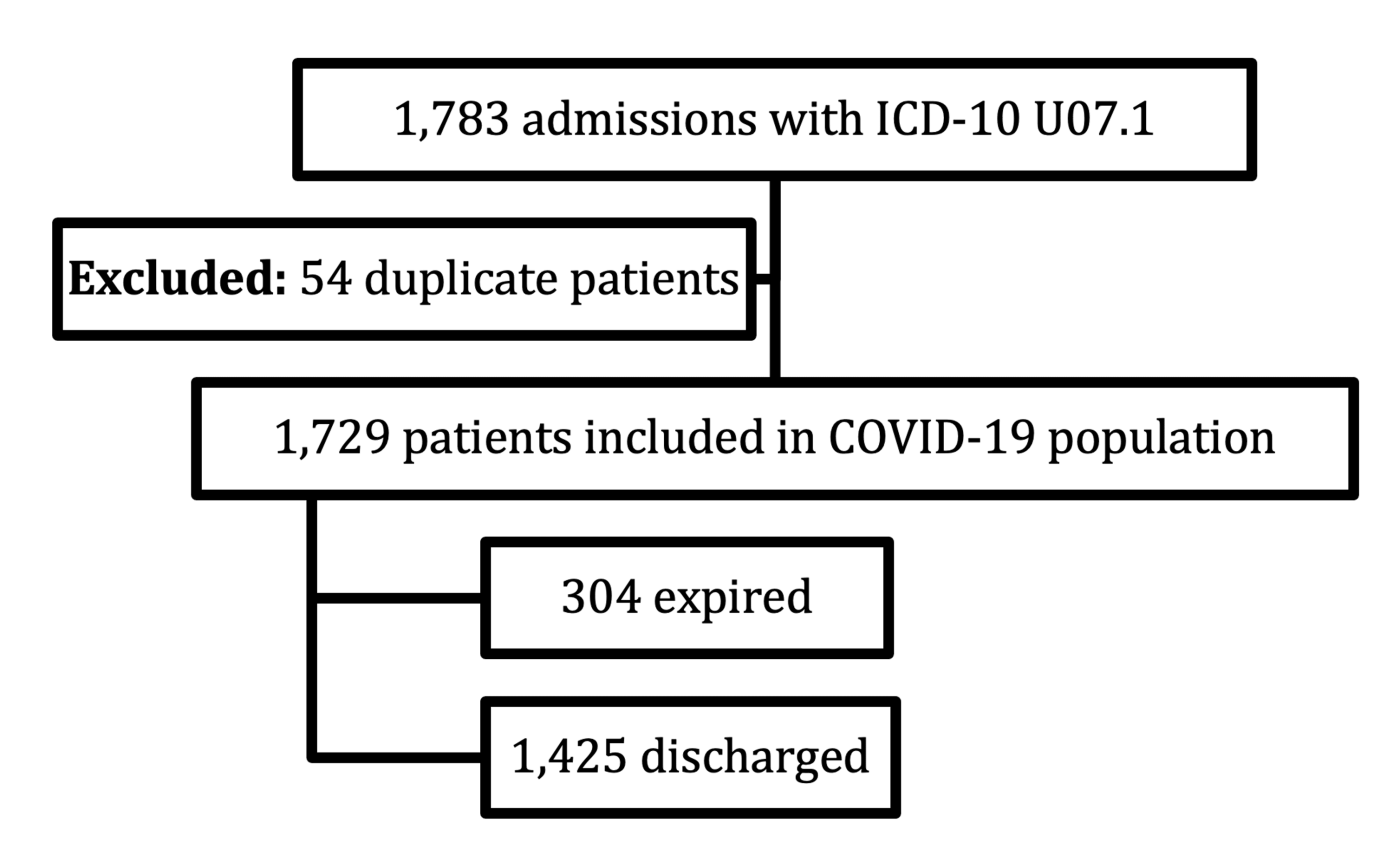
COVID-19 population and outcomes according to ICD-10 coding. ICD-10: International Classification of Diseases Tenth Revision

**Figure 2:**
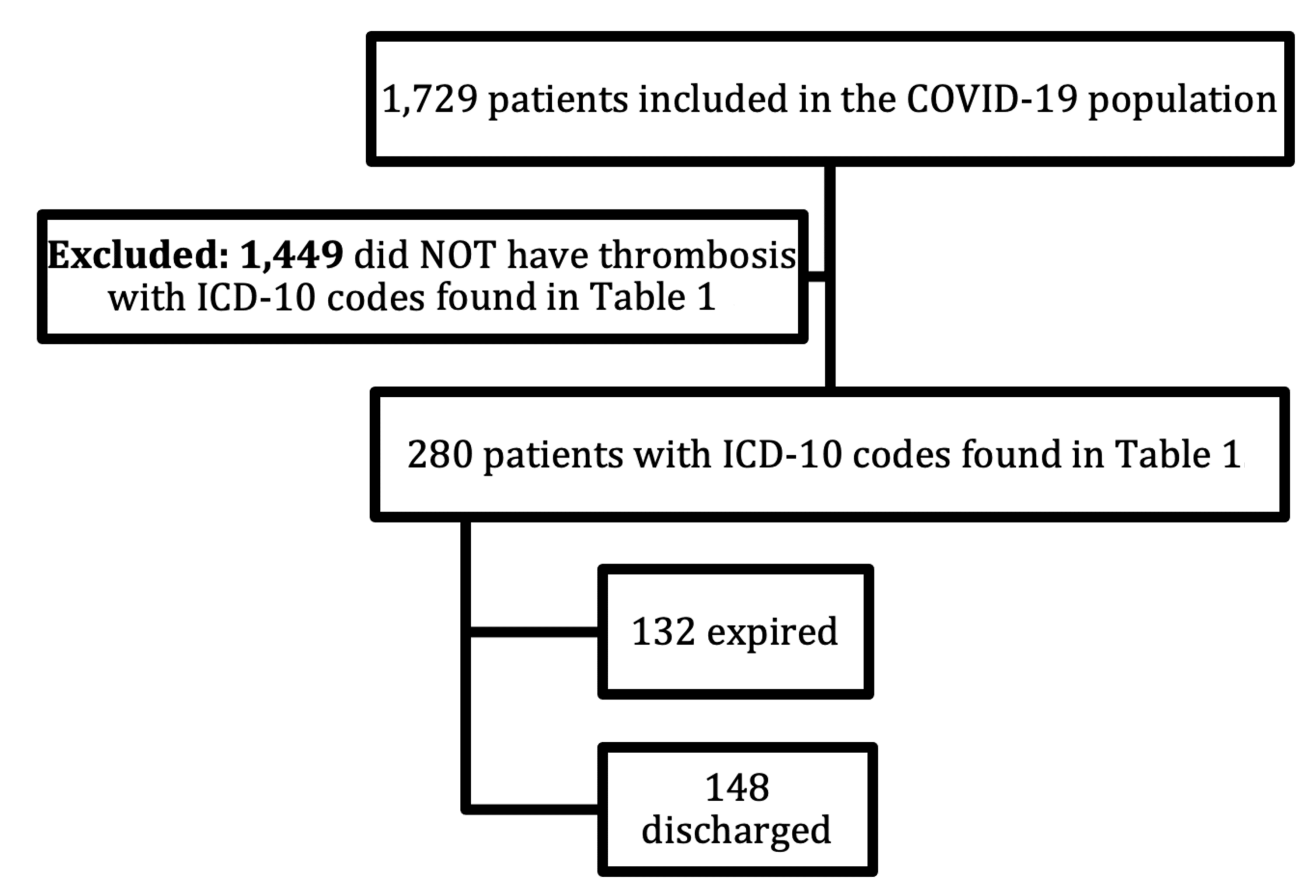
Thrombosis without COVID-19 diagnosis population. ICD-10: International Classification of Diseases Tenth Revision

During the study time frame, 1,783 patients were admitted with the COVID-19 ICD-10 code in Table 1. After excluding 54 duplicate admissions, 1,729 patients remained in the analysis. Of the 1,729 patients diagnosed with COVID-19, 304 expired and 1,425 survived to hospital discharge (Figure 1). Within the population diagnosed with COVID-19 there were 1,449 patients who did not have thrombosis, while 280 patients had thrombosis in addition to COVID-19 (Figure 2).

**Figure 3:**
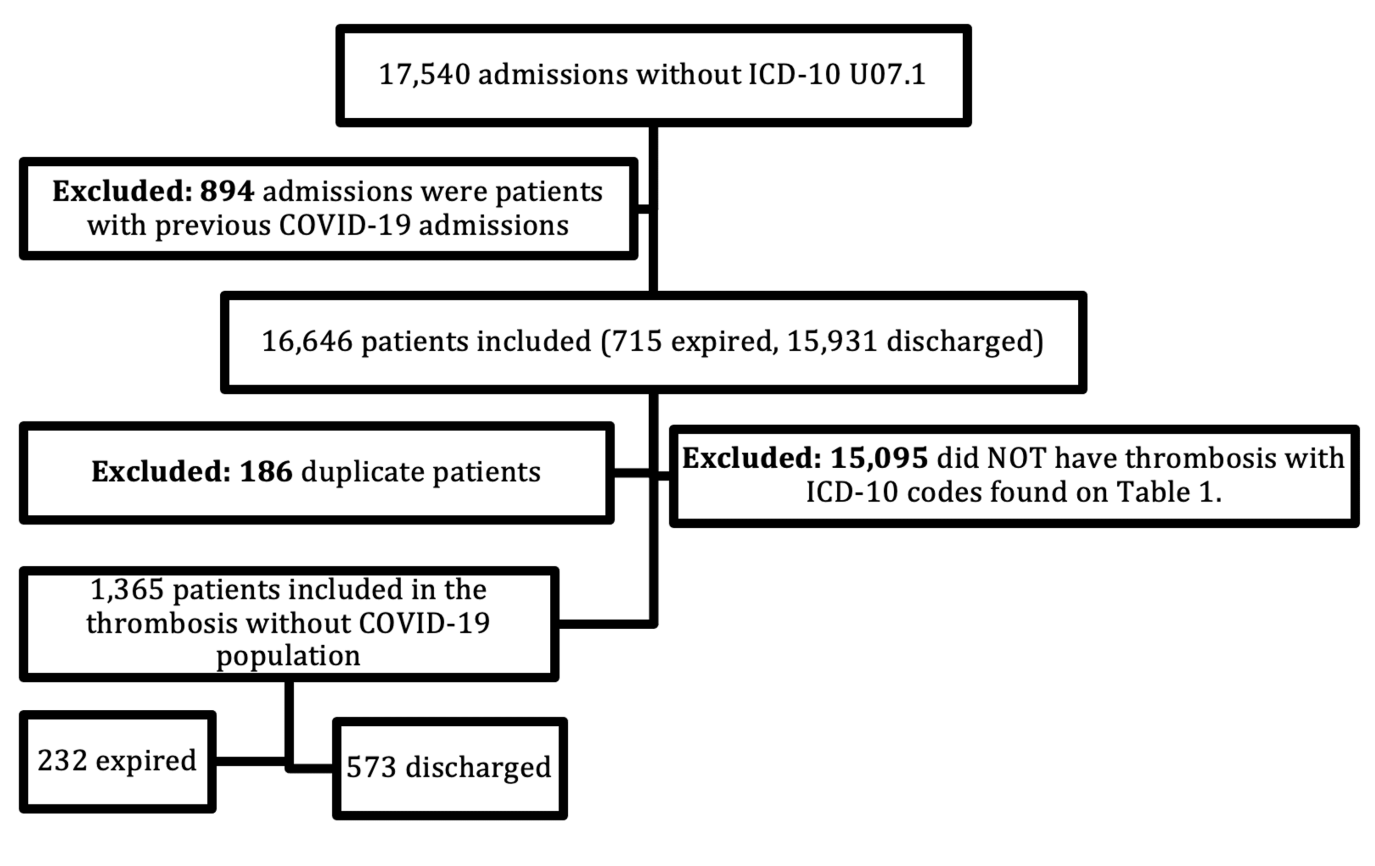
Thrombosis with COVID-19 combined population. ICD-10: International Classification of Diseases Tenth Revision

As seen in Figure 3, initially, there were 17,540 admissions to FHS without a COVID-19 diagnosis. Of these patients, 894 had been diagnosed with COVID-19 on previous admissions and were excluded from the study. Patients were excluded from this group based on previous COVID-19 admissions, which left 16,646 patients for consideration. Out of that number, 15,095 patients did not have the thrombosis ICD-10 codes in Table 1, and 186 duplicate patients were excluded. This left 1,365 patients with thrombosis but without the diagnosis of COVID-19.

From these three groups, statistical analysis based on mortality was performed on each of the following groups: thrombosis with COVID-19 (Population 1 {P1}), thrombosis without COVID-19 (Population 2 {P2}), and COVID-19 without thrombosis (Population 3 {P3}). The groups were further analyzed based on differences in biological sex - male or female - and age - above or equal to 65 years of age and below 65 years of age. There was no exclusion based on race or ethnicity; however, in Southwest Missouri, the patient population is predominately Caucasian. Sample proportions were completed using Wald’s method, and two sample proportion summary hypothesis tests with confidence intervals were used for the proportion difference.

## Results

A total of 3,094 patients were included in the study population utilizing ICD-10 codes shown in Table 1. These patients were separated into three groups and analyzed - 1,449 were diagnosed with COVID-19 without thrombosis (P2), 1,365 were diagnosed with thrombosis without COVID-19 (P3), and 280 were diagnosed with thrombosis and COVID-19 (P1) (Table 2).

**Table 2:** Mortality proportions within population groups. Individual group mortality sample proportions were produced using Wald’s method. P: Population

Populations	n	Mortality	Sample proportion	Lower 95% CI	Upper 95% CI
COVID-19 with thrombosis (P1)	280	132	0.4714	0.413	0.5299
COVID-19 without thrombosis (P2)	1449	172	0.1187	0.102	0.1354
Thrombosis without COVID-19 (P3)	1365	166	0.1216	0.1043	0.139

Patients from P3 who had previously been diagnosed with COVID-19 and duplicate patients from both P1 and prior COVID-19 admissions were excluded from the analysis. Comparing each of the three groups, age groups (≥65 and <65 years), and biological sex (M and F), there were no differences found. The most unbalanced group when considering these two factors was found within P1, with 62% of the group being male (Table 3).

**Table 3:** Characteristics within population groups. Stratified data of each population into biological sex, age, and race. P: Population

Characteristic	COVID-19 with thrombosis (P1)		COVID-19 without thrombosis (P2)		Thrombosis without COVID-19 (P3)		Full sample (3,094)	
	n	%	n	%	n	%	n	%
Gender								
Male	174	62	774	53	778	57	1726	56
Female	106	38	675	47	587	43	1368	44
Age (years)								
<45	24	9	199	14	147	11	370	12
45-59	69	25	362	25	298	22	729	24
60-75	123	44	529	37	635	47	1287	42
>75	64	23	359	25	285	21	708	22
Race								
Hispanic	17	6	51	4	20	1	88	2
Black or African American	5	2	25	2	17	1	47	2
Asian	1	<1	6	<1	4	<1	11	<1
Caucasian	242	41	1309	90	1274	93	2825	91
American Indian/Alaska Native	2	<1	14	1	15	1	31	1
Native Hawaiian or other Pacific Islander	9	3	24	2	12	<1	45	1
Other	4	3	20	1	23	2	47	2

Mortality within P1 was found to be significantly higher compared to P2 and P3. The mortality rate difference when comparing P1 to P2 and P3 was significant with a p<0.0001 (Table 4). There is 95% confidence that the mortality rate in P1, compared to P2, is between 29.19% and 41.35% higher. Similarly, there is 95% confidence that the mortality rate in P1, compared to P3, is between 28.88% and 41.08% higher. No significant difference was found when comparing biological sex or age concerning mortality rate. Within the analyzed populations, patients in P1 had an increased mortality rate when compared to those in P2 and P3.

**Table 4:** Mortality comparisons between population groups. Two sample proportion summary hypothesis tests with confidence intervals for the proportion difference. P: Population

Comparison	P1 (sample proportion)	P2 (sample proportion)	P1 and P2 sample proportion	Lower 95% CI for P1 and P2	Upper 95% CI for P1 and P2	p-Value
P1 vs P2	132 of 280 (0.4714)	172 of 1449 (0.1187)	0.3527	0.2919	0.4135	<0.0001
P1 vs P3	132 of 280 (0.4714)	166 of 1365 (0.1216)	0.3498	0.2888	0.4108	<0.0001
P2 vs P3	172 of 1449 (0.1187)	166 of 1365 (0.1216)	0.0029	-	-	0.8125

## Discussion

In this retrospective observational study, we compared mortality rates between patients hospitalized with COVID-19 and thrombosis (P1) and those hospitalized with COVID-19 without thrombosis (P2) and thrombosis without COVID-19 (P3). These groups were identified based on ICD-10 codes for COVID-19 and thrombosis. When comparing P1 vs P2 and P1 vs P3, there was a significant difference indicated by a p<0.0001. The statistical tests show that P1 has a greater mortality rate than P2 or P3 populations, indicating that patients diagnosed with COVID-19 and thrombosis had a higher likelihood of mortality compared to individuals diagnosed with COVID-19 and individuals diagnosed with thrombosis separately. This finding was expected as thromboembolisms can be a secondary complication to COVID-19 infection, worsening prognosis [5]. No difference in mortality was found between the P2 and P3 populations, supporting the conclusion that the combination of COVID-19 and thrombosis represents the most deadly condition in our study. There were no differences in mortality found between males and females within any of our population groups (COVID-19 with thrombosis, COVID-19 without thrombosis, and thrombosis without COVID-19). Surprisingly, in the population with COVID-19 and thrombosis, comparing the mortality rate in patients older than or equal to 65 years of age vs patients younger than 65 years of age, there were no differences found in trends of mortality. In a 2020 systematic review and meta-analysis, it was found that older age, sex, clinical severity assessed by using Sequential Organ Failure Assessment (SOFA) score, and high D-dimer were associated with mortality [10]. Though, age was not a factor associated with increased mortality in our population diagnosed with COVID-19 and thrombosis. However, the older populations had a higher mortality rate in both the COVID-19 without thrombosis population and the thrombosis without COVID-19 population. This was indicated by a significant difference in mortality when comparing those older than or equal to 65 years of age vs those younger than 65 years of age in both populations. The COVID-19 with thrombosis population had higher mortality regardless of age or biological sex.

There were several limitations to the study. The study population was acquired from one health system in Southwest Missouri, which could have different results from other populations outside the midwest or in urban and suburban areas. Our population was mainly Caucasian. Differences in results may exist in other ethnic groups. Also, this was a retrospective study; therefore, the sample was not chosen at random, and it is unable to be determined if the sample analyzed is representative of the population as a whole. It should also be considered that our COVID-19 and thrombosis population had a smaller sample size in comparison to the other populations in the study. Finally, the study did not account for the presence or absence of other comorbidities present within the study population. The strengths of our study exist in the fact that it is a retrospective analysis of a larger population when assessing the mortality of disease. Our study is reproducible as it requires access to a healthcare system and ICD-10 codes to perform statistical analysis, allowing reproduction at any time.

A common finding among COVID-19 patients is high D-dimer levels, associating to a worse prognosis [11]. In a study by Gómez-Mesa et al., it was concluded that using low molecular weight heparin as deep vein thrombosis (DVT) prophylaxis in the initial stages of COVID-19 infection reduced mortality by 48% at seven days and 37% at 28 days [7]. This indicates the detrimental effects of COVID-19 on clotting risk in the hospitalized populations. Proper initial anticoagulation prior to coagulopathies can lower the incidence of mortality in those with COVID-19 [12]. Monitoring hematologic labs for patients with COVID-19 is of great importance, as there is a higher risk of developing thrombosis and increasing the chances of mortality [13]. Though the choice of anticoagulation method is out of the scope of our study, it is a very important topic to discuss further for the treatment of admitted COVID-19 patients. Since the total mechanism is not completely understood, there is a need for greater research on this topic to prevent further mortality.

## Conclusions

The goal of the study was to determine the effect of thrombosis on mortality rates in a population diagnosed with COVID-19. Our data indicated that individuals diagnosed with both COVID-19 and thrombosis had a higher likelihood of mortality compared to those diagnosed with COVID-19 without thrombosis and those diagnosed with thrombosis without COVID-19.

The study goal was to determine the effect of thrombosis on mortality rates seen in a population diagnosed with COVID-19. Our data indicated that those diagnosed with COVID-19 and thrombosis had a higher likelihood of mortality when compared to populations diagnosed with COVID-19, without thrombosis and thrombosis, without COVID-19. This information could assist physicians in determining treatment plans for a patient diagnosed with COVID-19 and a secondary complication of thrombosis. Further studies could investigate the effects of thrombosis on COVID-19 on a larger scale to understand the trends in mortality in different populations.
